# Integrating Morphological, Molecular, and Climatic Evidence to Distinguish Two Cryptic Rice Leaf Folder Species and Assess Their Potential Distributions

**DOI:** 10.3390/insects17010126

**Published:** 2026-01-22

**Authors:** Qian Gao, Zhiqian Li, Jihong Tang, Jingyun Zhu, Yan Wu, Baoqian Lyu, Gao Hu

**Affiliations:** 1 State Key Laboratory of Agricultural and Forestry Biosecurity, College of Plant Protection, Nanjing Agricultural University, Nanjing 210095, China; 2 Environment and Plant Protection Institute, Chinese Academy of Tropical Agricultural Sciences, Haikou 571101, China; 3 Key Laboratory of Integrated Pest Management on Tropical Crops, Ministry of Agriculture and Rural Affairs, Haikou 571101, China; 4 Key Laboratory of Surveillance and Management of Invasive Alien Species, Guizhou Education Department, Department of Biology and Engineering of Environment, Guiyang University, Guiyang 550005, China

**Keywords:** *Cnaphalocrocis medinalis*, *Cnaphalocrocis patnalis*, climate change, population dynamics, MaxEnt, potential suitable region

## Abstract

The larvae and damage symptoms of *Cnaphalocrocis medinalis* and *Cnaphalocrocis patnalis* exhibit a high degree of similarity, which often leads to confusion between the two species. This has posed challenges for research on their population dynamics and the development of effective control measures. To better understand their morphological and damage characteristics, population dynamics, species identification based on *COI* gene fragments, and potential future distribution, a searchlight trap monitoring program was conducted for *C. medinalis* and its closely related species *C. patnalis* across four sites in Longhua, Haitang, and Yazhou districts in Hainan Province from 2021 to 2023. The MaxEnt model was utilized to predict the potential global distribution of both species, incorporating known occurrence points and climate variables. The trapping results revealed that both species reached peak abundance between April and June, with a maximum of 1500 individuals captured in May at Beishan Village, Haitang District. Interannual population fluctuations of both species generally followed a unimodal pattern. Genetic analyses revealed distinct differences in the mitochondrial *COI* gene fragment, confirming that *C. medinalis* and *C. patnalis* are closely related yet distinct species. The population peak of *C. patnalis* occurred slightly earlier than that of *C. medinalis*, and its field damage was more severe. Infestations during the booting to heading stages of rice significantly reduced seed-setting rates and overall yield. Model predictions indicated that large areas of southern Eurasia are suitable for the survival of both species, with precipitation during the wettest month identified as the primary environmental factor shaping their potential distributions. At present, moderately and highly suitable habitats for *C. medinalis* account for 2.50% and 2.27% of the global land area, respectively, whereas those for *C. patnalis* account for 2.85% and 1.19%. These results highlight that climate change is likely to exacerbate the damage caused by both rice leaf-roller pests, particularly the emerging threat posed by *C. patnalis*. Overall, this study provides a scientific basis for invasion risk assessment and the development of integrated management strategies targeting the combined impacts of *C. medinalis* and *C. patnalis*.

## Simple Summary

At present, infestations by *Cnaphalocrocis patnalis* Bradley (Lepidoptera: Crambidae) are showing an increasing trend, yet monitoring efforts remain primarily focused on *Cnaphalocrocis medinalis* Guenée (Lepidoptera: Pyralidae). In the context of ongoing climate change, reducing monitoring discrepancies between *C. medinalis* and *C. patnalis* is of critical importance for improving pest surveillance and addressing existing research gaps regarding these two species. Accordingly, the MaxEnt model was employed to predict and analyze the potential geographic distributions of *C. medinalis* and its closely related species *C. patnalis* based on known occurrence records and climatic variables. The model performance was ≥0.9, indicating “good fit” predictive precision. Morphological observations and population dynamics analysis demonstrated that the two species are morphologically similar. Genetic analysis revealed differences in the *COI* gene fragment, confirming that they are closely related species. Nevertheless, *C. patnalis* causes more substantial damage in the field compared to *C. medinalis*. Moreover, the difference in their peak occurrence times is minimal. The MaxEnt results suggest that the distribution of *C. medinalis* may be suitable in regions between 20° N and 40° N, especially in most parts of southern Eurasia, while *C. patnalis* may be suitable in coastal regions near the equator. The precipitation of the wettest month is the primary factor influencing their distribution. Under future climate change scenarios, although low-suitability areas are extensive, the suitable area for both species, particularly *C. patnalis*, reaches its peak in the SSP245 scenario, covering 2.87% of the total land area, indicating a potential risk of occurrence. Based on these findings, the implementation of monitoring and control measures for *C. medinalis* and *C. patnalis* is recommended to ensure the safe production of rice.

## 1. Introduction

Rice (*Oryza sativa* L.) is one of the most important staple crops worldwide; however, its production is often threatened by various insect pests [1]. Among these, the rice leaf folder, *Cnaphalocrocis medinalis* (Guenée, 1854) (Lepidoptera: Crambidae), is one of the most destructive species [2]. Its larvae feed on rice leaves, causing the greatest damage during the grain filling stage, which substantially reduces photosynthetic capacity and poses a serious threat to yield security [2]. The adult moth is a long-distance migratory pest characterized by high fecundity, high hatching and survival rates, and strong damaging capacity, resulting in its widespread distribution across rice growing regions worldwide [3,4,5]. *Cnaphalocrocis patnalis* (Bradley, 1981) (Lepidoptera: Crambidae), commonly known as the rice leaf folder, is a closely related species in the same genus. It exhibits morphological characteristics and feeding behavior similar to *C. medinalis*, is easily confused with it in the field, and has not yet been officially reported in China [3,6]. The larvae similarly feed on the upper epidermis and mesophyll of rice leaves, producing pale lesions that expand as larval development progresses and ultimately result in leaf whitening. Such damage markedly reduces photosynthetic efficiency and disrupts normal plant growth, and in severe cases may lead to plant death [7]. *Cnaphalocrocis medinalis* and *C. patnalis* exhibit broadly similar life-history strategies. In both species, eggs are typically laid singly on rice leaves; larvae are mainly nocturnal, feeding within longitudinally rolled leaves, and pupation occurs inside the leaf rolls, with the pupal stage generally lasting about one week. The complete life cycle includes egg, larval, pupal, and adult stages, with only minor differences reported between the two species, whereas reliable discrimination relies primarily on larval coloration and adult morphological traits rather than on developmental or behavioral characteristics [8,9].

Outbreaks of rice leaf folder infestations became increasingly prevalent in China after the 1970s, reaching major epidemic levels in 2003 and 2007. Together with other major rice diseases, the total affected area expanded to as much as 75 million hectares. From 1961 to 2010, the total areas affected by rice diseases and insect pests increased by 7.27-fold and 4.72-fold, respectively, compared with those recorded in the early twentieth century [6,10,11,12]. Light-trap monitoring has shown strong concordance with actual field occurrence in tracking rice leaf folder population dynamics, making light based trapping a critical and effective tool for pest surveillance and management [13].

Climatic oscillations throughout the Neogene period have profoundly influenced species’ geographical distributions and genetic structures, and understanding how climate change shapes species ranges has long been a central theme in biogeographical research. In particular, greenhouse gas emissions, largely driven by fossil fuel consumption, are widely recognized as one of the greatest environmental threats to the future of human societies [14,15,16,17]. A growing body of evidence indicates that changes in temperature and humidity, increasing frequencies of meteorological hazards and extreme climatic events, together with shifts in agricultural practices, are collectively reshaping the habitats of agricultural pests.These emerging trends highlight the necessity of applying species distribution models (SDMs) to identify and characterize potential suitable habitats [10,18,19]. Among the available SDMs, the maximum entropy (MaxEnt) model is one of the most widely used tools. By relying solely on presence-only occurrence records and associated environmental variables, MaxEnt generates optimized predictions of habitat suitability and produces spatially explicit maps of potential species distributions [20,21,22]. Simulations in MaxEnt are typically conducted under alternative greenhouse gas emission trajectories, and recent modeling frameworks increasingly incorporate the latest generation of scenario-based experiments, namely the Shared Socioeconomic Pathways (SSPs), which represent distinct future pathways of socioeconomic development and associated emissions [23]. In recent years, SDMs have been extensively applied to infer range shifts of animal species under climatic oscillations and to assess how ongoing environmental change may alter their potential distributional boundaries [16,24].

Rising temperatures have driven a northward expansion of the northern boundary of rice cultivation, thereby enlarging the potential damage range of leaf folder pests [25,26]. Previous studies have primarily focused on the migratory pathways and management strategies of these pests; however, information on their spatial distribution remains limited under increasingly complex climate change scenarios, particularly for the rarely documented species *C. patnalis* [11,27]. Notably, Zhao et al. [11] employed the MaxEnt model in conjunction with GIS tools to predict the potential distribution of *Cnaphalocrocis medinalis* in China under climate change scenarios. However, their analysis was based on earlier climate frameworks and did not incorporate the most recent climate projections derived from the Shared Socioeconomic Pathways (SSPs). Consequently, it is essential to assess how future climate change may reshape the potential distributions of *C. medinalis* and *C. patnalis*, with particular emphasis on shifts in high risk regions. The innovative aspects of this study are clearly defined. First, this study presents the first global scale comparison of the potential distributions of *C. medinalis* and *C. patnalis*. Second, it provides the first systematic characterization of larval and adult morphological traits, together with an integrated *COI* based diagnostic approach. Finally, four future climate scenarios (SSP126–585) are employed to predict long-term differences in distributional risk under alternative climate change pathways.

Hainan Province is characterized by exceptionally favorable climatic conditions. The continued expansion of national seed breeding bases in the region, together with shifts in land-use patterns, has accelerated the spread of certain agricultural pests, thereby posing potential threats to local ecological security and, more broadly, to agricultural production at the national scale [28,29]. Ongoing climate warming has shortened the generational duration of *C. medinalis* to varying extents; however, its closely related species, *C. patnalis*, has been only sporadically recorded across the Eurasian continent. This situation highlights the urgent need to strengthen population dynamic monitoring to address the potential increase in the dominance of *C. patnalis* and the growing ecological complexity arising from its co-occurrence with *C. medinalis* [3,30]. Given Hainan Province’s proximity to current outbreak centers of these two rice leaf folder pests, as well as its favorable climatic and geographic conditions, we conducted searchlight trap monitoring at four sites in Haikou and Sanya from 2021 to 2023, providing the first detailed assessment of *C. patnalis* population dynamics in Hainan. Furthermore, by integrating known occurrence records with current climatic conditions, we applied the MaxEnt model for the first time to evaluate the potential global habitat suitability of *C. medinalis* and *C. patnalis*. These analyses offer important insights into their occurrence patterns and developmental dynamics and provide a scientific basis for improving monitoring, early-warning systems, and preventive management strategies in rice production systems.

## 2. Materials and Methods

### 2.1. Population Data Collection

**Figure 1:**
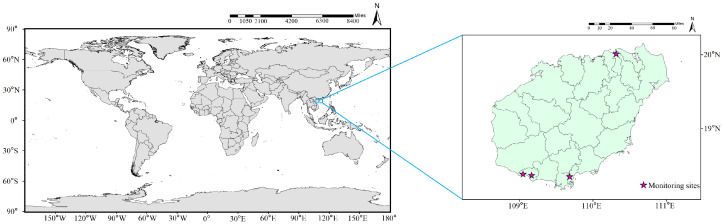
Schematic map of the study area and sampling sites.

From 2021 to 2023, field monitoring of migratory moth populations was conducted using searchlight traps at four locations across Hainan Province: Longhua District, Haikou City (20°1′ N, 110°19′ E), Beishan Village, Haitang District (18°21′ N, 109°41′ E), Dongcheng First Village in Yazhou District (18°22′ N, 109°10′ E) and Sangeng Village in Yazhou District (18°23′ N, 109°3′ E) (Figure 1). A total of four searchlight traps were deployed across these sites, providing spatial replication. Monitoring was conducted continuously over three consecutive years, providing temporal replication. Each searchlight trap consisted of a GT75 searchlight equipped with a ZJD 1000W metal halide lamp (Renco Measurement and Control Technology Co., Ltd., Weifang, China), designed to sample high-altitude insect migrants at elevations of up to approximately 500 m above ground level. The light source was mounted on an iron ring frame above a large galvanized iron funnel, with the lower opening fitted with a 20 cm diameter collecting port [31]. A rearing net (100 × 100 × 120 cm) attached to the bottom of the funnel was used to collect insects attracted during nocturnal operation. The traps were operated nightly and switched off at 06:00 the following morning, after which the collecting nets were retrieved and transferred to the laboratory. Captured insects were collected using 60 mesh nylon nets and briefly frozen at −20 °C to ensure rapid killing. Specimens were subsequently sorted and identified based on morphological characteristics. The resulting abundance data were used for analyses of seasonal population dynamics [32].

All specimens were identified by an experienced taxonomic expert and independently verified by a second specialist to ensure taxonomic accuracy. To further validate the reliability of morphological identifications, a randomly selected subset of specimens was subjected to molecular identification, which showed complete concordance with the morphological results. Population abundance data were analyzed using one-way analysis of variance (ANOVA) to assess interannual differences. When significant effects were detected, post hoc mean comparisons were conducted at a significance level of *p* < 0.05. All statistical analyses were performed using SPSS (version 27.0.1) to ensure analytical consistency. Raw datasets were curated and processed in Microsoft Excel, and all figures were generated using Origin, ensuring transparency and reproducibility of data processing and visualization workflows.

### 2.2. Insect Samples

Larvae of *C. medinalis* and *C. patnalis* were collected from rice fields in Sanya, Hainan Province (18°23′ N, 109°10′ E), in June 2025. Species identification was initially based on larval specimens obtained during field surveys. A total of ten individuals were collected. Genomic DNA was extracted from the larval samples using a commercial DNA extraction kit (Tiangen Biotech Co., Ltd., Beijing, China). The quality of the extracted DNA was assessed by agarose gel electrophoresis, and DNA concentration was measured using a microvolume spectrophotometer (Aurora 900, Seattle, WA, USA). Purified DNA samples were stored at −20 °C until further analysis.

### 2.3. PCR and Sequencing

The *COI* gene was amplified using a pair of universal primers (LCO1490-F, 5′-GGTCAACAAATCATAAAGATATTGG-3′, and HCO2198-R, 5′-TAAACTTCAGGGTGACC AAAAAATCA-3′), designed based on the FOLMER, were used to amplify the mitochondrial of *C. medinalis* and *C. patnalis*, with the extracted DNA samples serving as templates. PCR mixtures contained 1 μL of template DNA, 1 μL of forward primer, 1 μL of reverse primer, 12.5 μL of 2× Taq PCR MasterMix II, and ddH_2_O to a final volume of 25 μL. The amplification program consisted of an initial denaturation at 94 °C for 5 min, followed by 35 cycles of denaturation at 94 °C for 30 s, annealing at 55 °C for 50 s, and extension at 70 °C for 60 s, with a final extension at 72 °C for 10 min. An aliquot of 1 μL of the PCR product was subjected to electrophoresis on a 2% agarose gel for quality assessment. Qualified PCR products were subsequently sent to Qingke Biotechnology for Sanger sequencing (Beijing, China).

### 2.4. COI Sequence Alignment

Sequencing chromatograms were examined using SnapGene software (version 6.0.2). Low-quality regions at both ends of the sequences were trimmed to obtain high-quality gene fragments. The resulting sequences were then subjected to homology analysis using the BLAST tool on the NCBI website (BLAST, https://blast.ncbi.nlm.nih.gov/Blast.cgi?PROGRAM=blastn&PAGE_TYPE=BlastSearch&LINK_LOC=blasthome, accessed on 19 January 2026).

### 2.5. Global Occurrence Data of C. medinalis and C. patnalis

**Figure 2:**
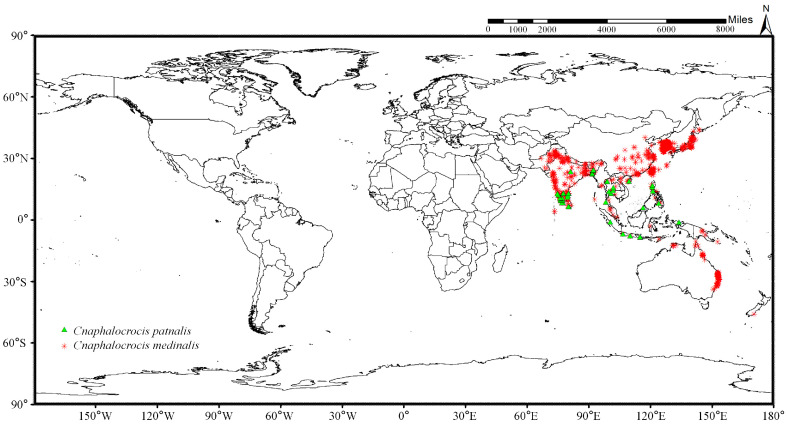
Global distribution maps of *C. medinalis* and *C. patnalis*.

The world wide distribution record data of *C. medinalis* and *C. patnalis* were obtained from the Global Biodiversity Information Facility (GBIF), on 15 November 2025 (https://doi.org/10.15468/dl.bht7tp; https://doi.org/10.15468/dl.x2qhq4). Only records with precise geographic coordinates were retained in the initial dataset. Following rigorous screening and quality assessment, selected records from public biodiversity databases were incorporated into the analysis. Although platforms such as iNaturalist, CABI, and EPPO provide extensive global distribution information, many records are derived from citizen-science observations or country-level presence reports. Given the high morphological similarity between *C. medinalis* and *C. patnalis* and the associated risk of misidentification in public databases, the final dataset was restricted to taxonomically validated occurrence records with accurate geographic coordinates, thereby minimizing classification errors in subsequent ecological niche modeling. The obtained distribution point data were then sparsified using the spThin package in R (version 4.5.2) software to ensure that only one distribution point was retained within each 5 km × 5 km range [33]. Finally, a total of 1657 distribution record points of *C. medinalis* and *C. patnalis* were obtained for the operation and verification of the MaxEnt model (Figure 2).

### 2.6. Bioclimatic Variables

In this study, 19 historical global bioclimatic variables were downloaded from the Worldclim database (https://www.worldclim.org/) with a spatial resolution of 5 arc-minutes and time range of 1970–2000 [34]. Future climate data were obtained from Coupled Model Interaction Project Phase 6(CMIP6) under the Beijing Climate Center Climate System Model 2 Medium Resolution (BCC–CSM2–MR) climate model for SSP126, SSP245, SSP370 and SSP585, which included the projection for the periods 2021–2040 (2030s), 2041–2060 (2050s), 2061–2080 (2070s),and 2081–2100 (2090s) [3,35,36]. These bioclimatic variables closely affected the growth and development of *C. medinalis* and *C. patnalis*. To avoid the influence of autocorrelation from multiple linear repeats among the extracted climate variables and avoid the overfitting of MaxEnt [37], screening and removal of some of the climate variables were performed to reduce the influence of redundancy on the prediction results. Firstly, redundant variables were excluded based on Pearson correlation coefficients (|r|≥0.8). For highly correlated variable pairs, variables with clearer ecological relevance to the biological processes of leaf-folder moths were preferentially retained. Ultimately, the five most influential environmental variables, ranked by percent contribution (Table S1), were selected separately for *C. medinalis* and *C. patnalis* for subsequent distribution predictions under both current and future climate scenarios. Ultimately, five bioclimatic variables (BIO3, BIO8, BIO13, BIO16, and BIO18) were selected for *C. medinalis*’ model construction. BIO3 (isothermality) represents the relationship between diurnal and annual temperature variability, capturing temperature stability rather than absolute temperature magnitude. BIO8 (mean temperature of wettest quarter) reflects thermal conditions during periods of high moisture availability, which are particularly important for species growth and survival. BIO13 (precipitation of wettest month), BIO16 (precipitation of wettest quarter), and BIO18 (precipitation of warmest quarter) characterize precipitation intensity and seasonal moisture availability during biologically active periods. Five bioclimatic variables (BIO1, BIO2, BIO3, BIO11, and BIO13) were ultimately retained for constructing the *C. patnalis* distribution model. BIO1 (annual mean temperature) reflects the general thermal conditions of the study area, whereas BIO2 (mean diurnal range) describes short-term temperature fluctuations. BIO3 represents the relative magnitude of diurnal temperature variation compared with annual temperature range, indicating temperature stability. BIO11 (mean temperature of the coldest quarter) captures thermal constraints during the coldest period of the year, which may limit species survival. BIO13 characterizes moisture availability during periods of maximum rainfall.

Climatic variable values corresponding to occurrence points were extracted from the WorldClim datasets using the spatial analysis tools in ArcGIS (version 10.8). WorldClim data were selected to characterize the macroclimatic constraints on the potential global distributions of *C. medinalis* and *C. patnalis*. Although fine-scale microclimatic variation may strongly influence short-term population dynamics at local scales, such processes cannot be explicitly captured by global species distribution models. Therefore, microclimatic effects inferred from field monitoring data were not directly incorporated into the MaxEnt models, allowing a clear distinction between macro-scale habitat suitability and local population fluctuation dynamics.

### 2.7. MaxEnt Model Setting and Selection

Models were evaluated using spatially independent block partitioning, and the optimal model was selected based on the lowest Akaike Information Criterion corrected for small sample sizes (AICc). The final model employed linear features (L) with a regularization multiplier of 2. The parameters of the MaxEnt model were set as follows: the model output format was selected as “Logistic”, file type as “Asc”, and replication run type as “Subsample”. To identify the most appropriate set of feature classes (FCs) for the MaxEnt model, we followed a systematic process that considered both model complexity and ecological relevance. Initially, we tested five commonly used FC combinations: Linear (L), Quadratic (Q), Linear + Quadratic (LQ), Linear + Quadratic + Hinge (LQH), and Hinge (H). These combinations were selected to capture a range of potential ecological responses, from simple linear relationships to more complex non-linear and threshold-based interactions. A range of regularization multipliers (1–5) was evaluated to balance model fit and overfitting. We used the “ENMeval” package in R software to calculate the FC and AICc [38,39,40]. The difference between the training and test areas of the receiver operating characteristic curve ($AUC_{diff}$) and AICc was used to select the optimal parameter combination for the MaxEnt model [39]. For background point selection and environmental data extraction, 10,000 background points were randomly sampled within the species’ accessible area (M). The accessible area was defined as the union of 50-km buffers surrounding all occurrence records, representing the environmental space potentially accessible to the species under historical dispersal and ecological constraints.

### 2.8. MaxEnt Model Evaluation and Analysis

We evaluated MaxEnt model performance using the receiver operating characteristic (ROC) curve and the area under the curve (AUC). As a threshold-independent metric, AUC reflects the model’s ability to discriminate occurrence records from background locations. The range of the AUC is from 0 to 1, and the classification used to assess the accuracy of the MaxEnt results was failing (0–0.6), poor (0.6–0.7), fair (0.7–0.8), good (0.8–0.9), and excellent (0.9–1.0) [41,42,43]. The prediction results for both current and future scenarios were imported into ArcGIS using the ASCII to Raster tool and reclassified into four risk levels based on the Natural Breaks (Jenks) method. Subsequently, the Reclassify tool was applied to the MaxEnt output maps to quantify the geographic extent of suitable habitats, with values ranging from 0 to 1, where higher values indicate a higher probability of species presence [44]. For *C. medinalis* and *C. patnalis* habitat suitability were divided into four classes: unsuitable region (0–0.06), lowly suitable region (0.06–0.21), moderately suitable region (0.21–0.42), and highly suitable region (0.42–1.00) [45].

### 2.9. MaxEnt Model Optimization and Accuracy Evaluation

Although more complex feature classes were tested, linear features provided the optimal balance between performance and complexity. In this study, the performance of the optimized MaxEnt model is excellent. After the optimization, the model parameters in this study were RM=2, FC=L, $AUC_{\mathrm{diff}\left(\mathrm{avg}\right)}=0.066$, and $\Delta AIC_{c}=0$. MaxEnt was then used to predict the current potential distribution of *C. medinalis* and *C. patnalis*. Each model was run 10 replicate times, with 75% of the occurrence records randomly selected for model training and the remaining 25% used for model testing, ensuring robust model performance and realistic representation of species distribution probabilities [45]. The proportion of test data was defined using a random seed, and the replicated run type was set to subsample. The maximum number of iterations was set to 5000. The importance of climatic variables was assessed using a jackknife test, and the effects of individual variables on the potential distributions of *C. medinalis* and *C. patnalis* were examined by generating response curves. Model outputs were produced in logistic format, and all other parameters were left at their default settings [46]. The proportional areas of suitable habitats were calculated under current climatic conditions and across the four future SSP scenarios. These results were visualized using pie charts, which were presented alongside the corresponding suitability distribution maps. A consistent legend was applied across all suitability classifications to facilitate direct comparison among scenarios and between species.

## 3. Results

### 3.1. Morphological Differences

**Figure 3:**
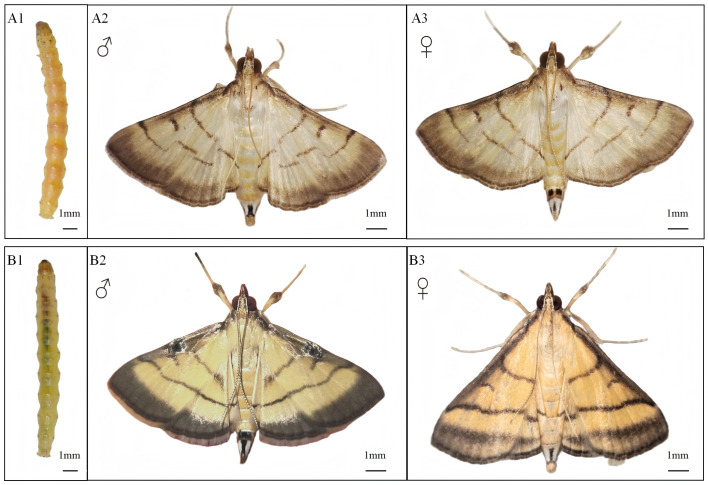
Morphological Differences between *C. patnalis* and *C. medinalis*. (**A1**–**A3**) *C. patnalis*; (**B1**–**B3**) *C. medinalis*.

The key distinguishing morphological characteristics of the two leaf roller larvae are illustrated in Figure 3(A1,B1)). Larvae of *C. medinalis* (Figure 3(B1)) possess a green body and a yellowish brown head capsule bearing brown setae. The prothoracic shield displays a pair of brown spots that extend into bracket shaped markings. Both the mesothoracic and metathoracic shields bear eight circular spots, each characterized by a black outer ring and a brown central area. The spiracles are distinctly black, and the body coloration gradually darkens toward the posterior end, likely reflecting chlorophyll accumulation during later feeding stages [47].

In contrast, larvae of *C. patnalis* (Figure 3(A1)) exhibit a yellowish brown to pale yellow body with a relatively translucent integument marked by fine punctures and sparse brown setae. The head capsule is noticeably paler than that of *C. medinalis*. Abdominal spiracles are prominent and black, and the larvae appear generally smooth, with clearly defined intersegmental boundaries.

The principal morphological differences between adults of *C. medinalis* and *C. patnalis* are illustrated in Figure 3(A2,A3,B2,B3). Adults of *C. medinalis* (Figure 3(B2,B3)) possess a light yellowish brown to pale brown body and a relatively slender form. The forewings are characterized by a dark brown anterior margin and a conspicuous broad brownish black outer margin. Three dark brown transverse lines, inner, median, and outer, are present on the forewing surface, with the median line shorter and not extending to the posterior margin. The hindwings likewise display a broad brownish black outer margin. Both forewings and hindwings have a pale yellowish brown ground color with two horizontal dark bands and a glossy appearance.

Pronounced sexual dimorphism is evident in *C. medinalis*. Males possess a round, slightly recessed, glossy spot located at the midpoint of the anterior margin of the forewing, commonly referred to as an “eye spot” which is absent in females. This feature serves as a key diagnostic character for sex differentiation.

In contrast, *C. patnalis* adults exhibit three distinct transverse lines on the forewings (Figure 3(A2,A3)). The median line extends only to the anterior margin of the median cell, whereas the outer line curves inward toward the center before extending downward. On the hindwings, the inner transverse line is longer than the outer one. Sexual differences are also evident in the terminal morphology: females have a blunt posterior end bearing a rectangular black spot on the dorsal surface, whereas males possess a pointed posterior end with two small, square black spots dorsally.

### 3.2. Damage Symptoms

**Figure 4:**
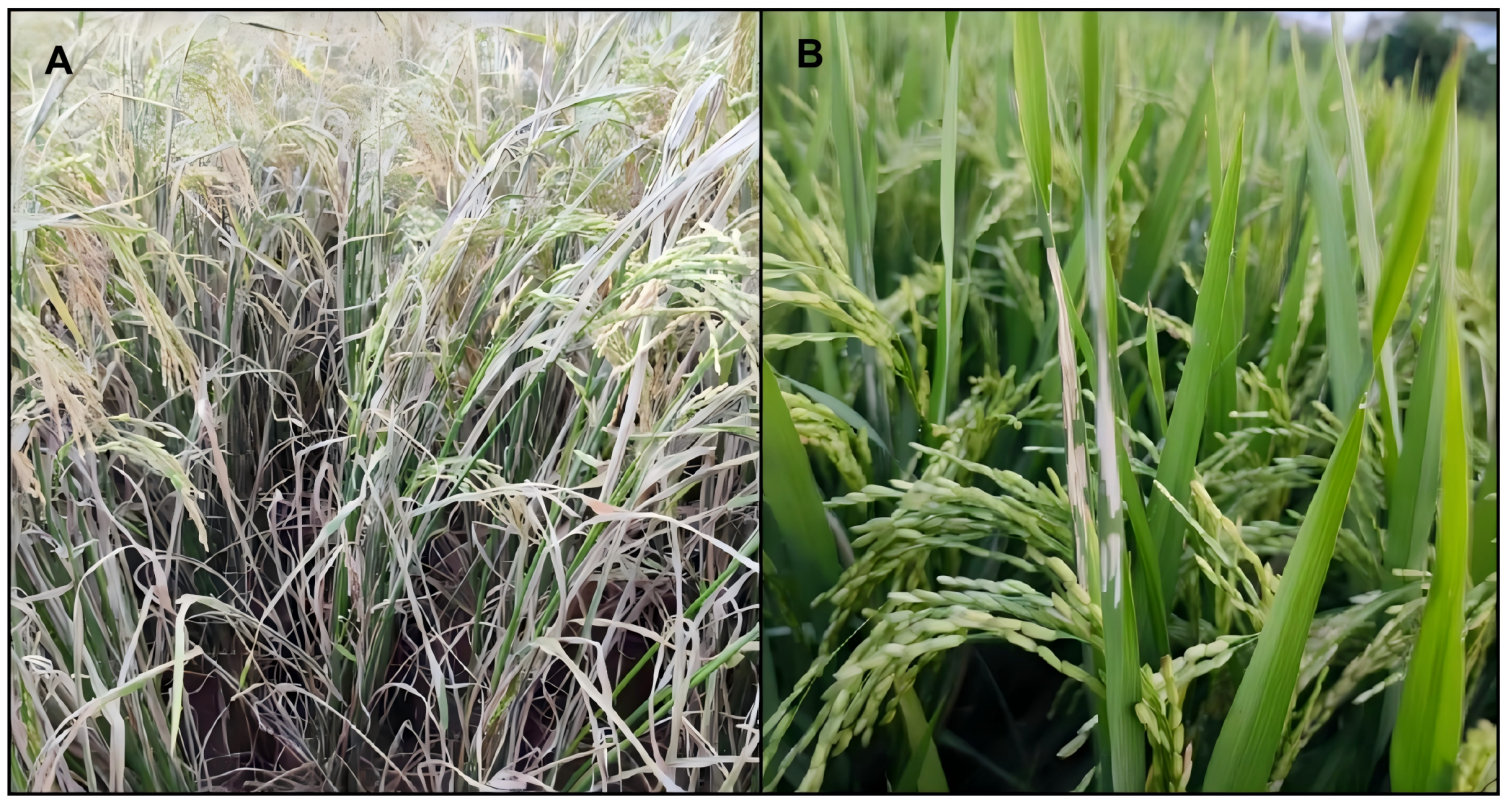
Damage symptoms on rice plants caused by *C. patnalis* and *C. medinalis*. (**A**) *C. patnalis*, (**B**) *C. medinalis*.

The larvae of *C. patnalis* primarily feed on the leaves of rice plants. Newly hatched larvae initially consume the heart leaves, leaving small, pinpoint sized white dots on the affected leaf surface. Some larvae begin feeding inside the leaf sheath. As the larvae mature, the damaged leaves develop irregularly shaped white stripes, which extend from the leaf margin toward the leaf surface, eventually curling into a tubular form. This results in the leaves being rolled into a longitudinal fold, with the larvae concealed within. They feed on the leaf epidermis and mesophyll, leaving behind a white, remaining lower epidermis. Damage symptoms vary across rice growth stages. Injury during the seedling stage suppresses plant growth and may even cause plant death under severe infestation. From the tillering to jointing stages, damage results in reduced tiller numbers, shorter plant height, and delayed development. During the panicle stage, particularly from heading to full heading, injury to the flag leaf is most apparent, leading to reduced flowering and grain set, increased spikelet sterility, and decreased thousand grain weight. Among these stages, damage occurring during panicle initiation and heading causes the greatest yield losses. Under severe infestation, all leaves may become tightly rolled and desiccated, which severely impairs photosynthesis and grain filling. The level of damage can exceed that caused by *C. medinalis* (Figure 4A).

### 3.3. Monitoring Seasonal Population Dynamics

**Figure 5:**
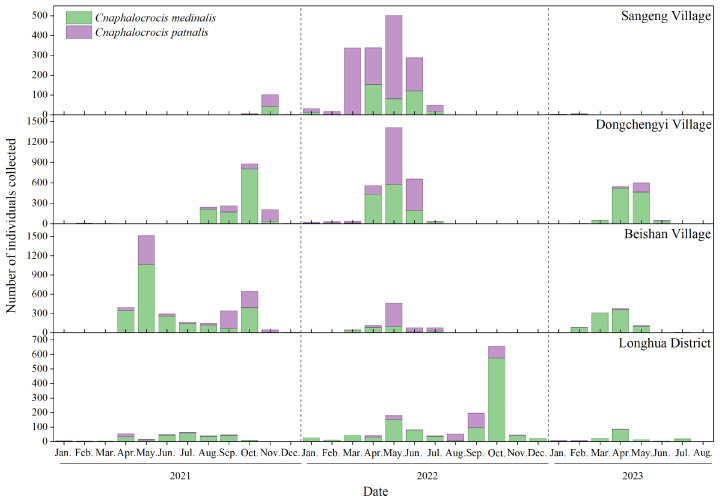
Dynamics of the number of *C. medinalis* and *C. patnalis* trapped using searchlight traps in different months and locations.

**Figure 6:**
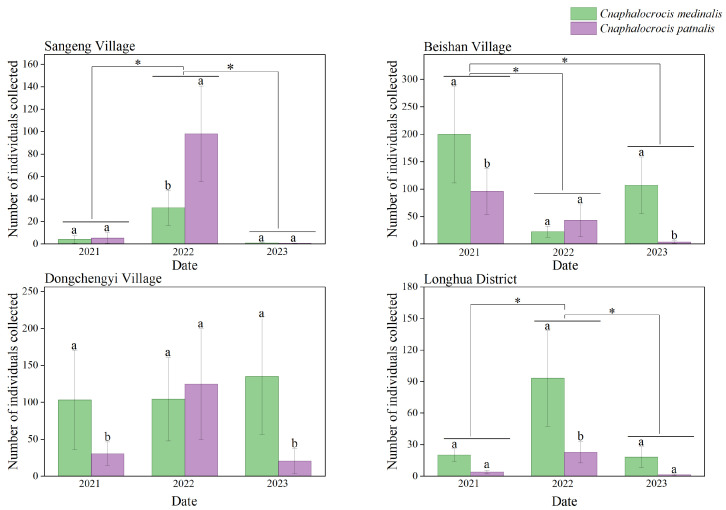
Analysis of the dynamics of the number of *C. medinalis* and *C. patnalis* trapped using searchlight traps in different years and locations. Note: Significant differences in the total occurrence of the two leaf-roller species among different years are indicated by “*” (*p* < 0.05). Lowercase letters denote significant differences (*p* < 0.05) in the occurrence of the two species within the same year.

The spatial distribution of the filtered occurrence records for *C. medinalis* and *C. patnalis* is shown in Figure 2. These records are primarily concentrated in the Eurasian region, spanning from the Equator to the Tropic of Cancer, including areas such as Korea, Japan, the Philippine archipelago, and the eastern coastal regions of Australia. Hainan Province was selected as the experimental site due to its tropical climate and intensive rice cultivation, which provide highly suitable conditions for both species, making it an appropriate and representative region for this study. From 2021 to 2023, searchlight traps were installed at four experimental sites across Hainan Province to monitor the population dynamics of *C. medinalis* and *C. patnalis*. The results revealed distinct seasonal fluctuations and regional differences in the populations of these two species across different years and locations (Figure 5). Both species exhibited major outbreaks in 2022, during which trap catches at all monitoring sites were substantially higher than in 2021 and 2023 (Figure 6). For *C. medinalis*, population peaks occurred between April and June 2022. The highest captures were recorded at Dongcheng First Village, reaching 573 individuals in May. Sangeng Village also showed marked peaks, with 153 and 120 individuals captured in April and June, respectively. The population peaks of *C. patnalis* were largely synchronous with those of *C. medinalis*, although in some regions (e.g., Dongcheng First Village and Longhua District) peaks occurred slightly earlier. At Dongcheng First Village, *C. patnalis* reached its maximum trap catch in May, with 836 individuals recorded. Based on data from all monitoring sites, both *C. medinalis* and *C. patnalis* began to appear after March as temperatures increased, entered a high incidence period from April to June, and declined rapidly after July, with populations nearly disappearing after August. This pattern represents a typical unimodal seasonal fluctuation. However, in 2021, a second population peak was observed from August to October, particularly at Dongcheng First Village and Beishan Village, indicating a bimodal pattern of population dynamics.

### 3.4. Identification of C. medinalis and C. patnalis Based on COI Fragments

**Figure 7:**
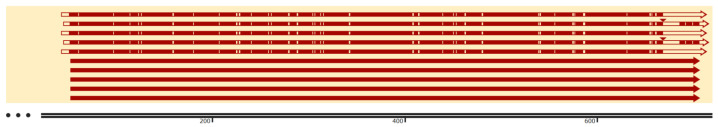
*COI* Sequences comparison between *C. medinalis* and *C. patnalis*. The upper five rows represent *C. patnalis*, and the lower five rows represent *C. medinalis*.

**Figure 8:**
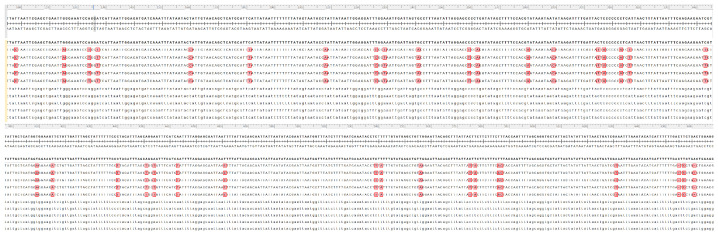
Diagnostic nucleotide Sites in the *COI* Gene of *C. medinalis* and *C. patnalis*. The upper five rows represent *C. patnalis*, and the lower five rows represent *C. medinalis*.

PCR amplification using *COI* primers showed excellent performance across all 10 samples, producing clear, single bands of the expected size. The amplified *COI* gene fragments were approximately 700 bp in length, consistent with theoretical expectations. Following sequencing and chromatogram correction, high quality and accurate *COI* sequences were obtained for all samples (Figure 7). Sequence alignment of the *COI* gene fragments from *C. medinalis* and *C. patnalis* identified a total of 37 variable sites within the 700 bp region, comprising 35 nucleotide substitution sites and 2 nucleotide deletion sites (Figure 8).

### 3.5. Global Potential Distribution

**Figure 9:**
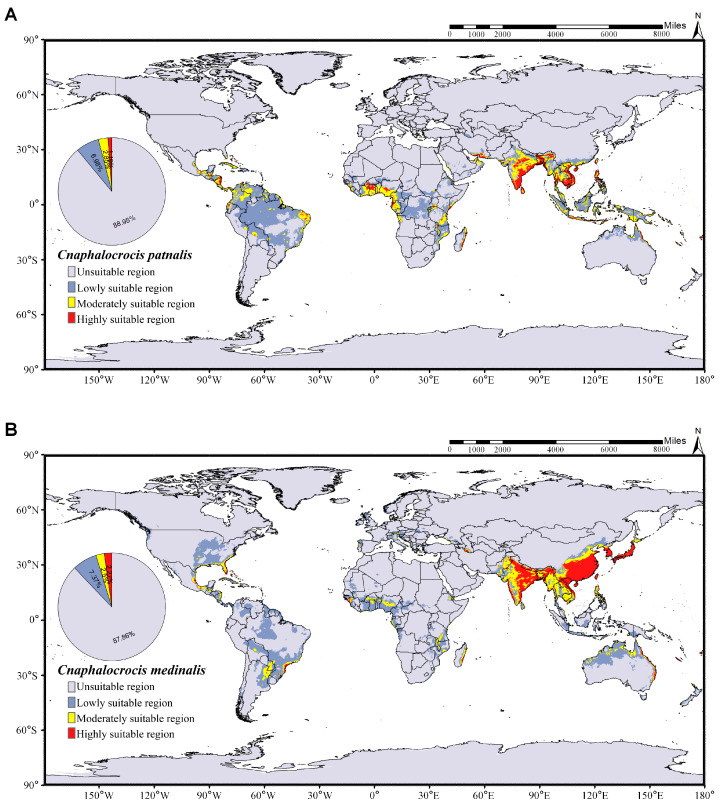
Predicted global habitat suitability for (**A**) *C. patnalis* and (**B**) *C. medinalis* under baseline climate (1970–2000).

**Figure 10:**
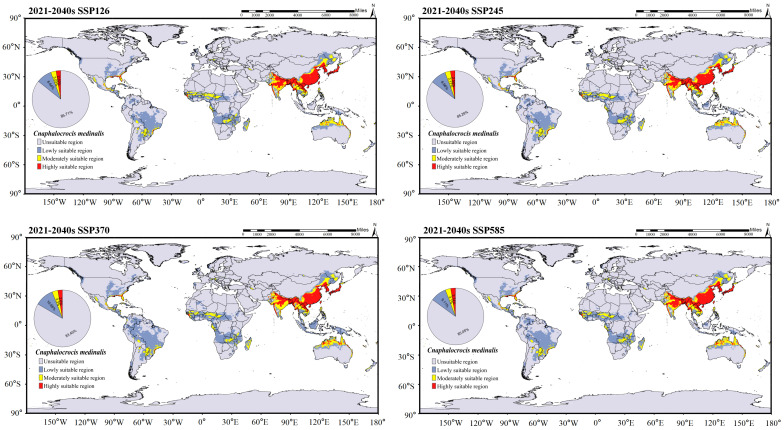
Distribution prediction of *C. medinalis* under different climate scenarios for the 2030s.

**Figure 11:**
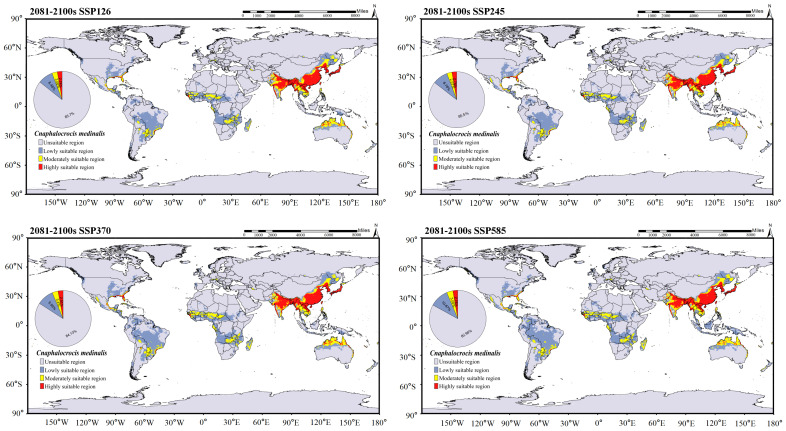
Distribution prediction of *C. medinalis* under different climate scenarios for the 2090s.

**Figure 12:**
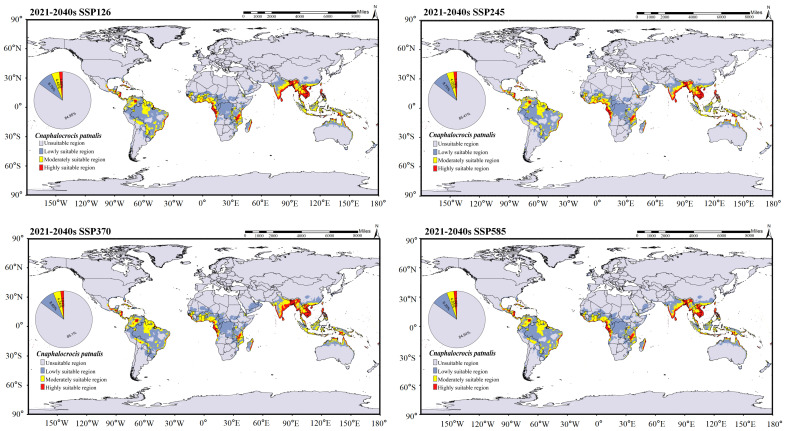
Distribution prediction of *C. patnalis* under different climate scenarios for the 2030s.

**Figure 13:**
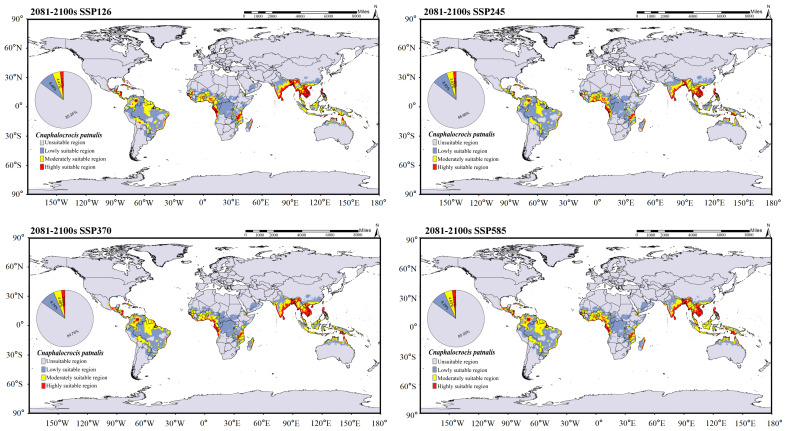
Distribution prediction of *C. patnalis* under different climate scenarios for the 2090s.

The global suitable distribution areas of *C. medinalis* and *C. patnalis*, as predicted by the MaxEnt model, are shown in Figure 9. Under current climatic conditions, the suitable habitat of C. medinalis extends across southern South America, central and southern Africa, the southern Eurasian continent, and northern Australia (Figure 9B). Moderately and highly suitable areas are mainly concentrated in the southern part of Eurasia, accounting for 4.77% of the total suitable area. The suitable distribution of *C. patnalis* encompasses northern South America, central Africa, and the southern Eurasian continent. Moderately and highly suitable habitats account for 4.04% of the total suitable area (Figure 9A). To assess the potential impacts of climate change, we further projected the future suitable distribution areas of *C. medinalis* and *C. patnalis* for the 2030s, 2050s, 2070s, and 2090s under four Shared Socioeconomic Pathway scenarios (SSPs: SSP126, SSP245, SSP370, and SSP585), as illustrated in Figure 10, Figure 11, Figure 12 and Figure 13 and S3–S6.

In the predicted suitable area of *C. medinalis* (Figure 10, Figure 11, Figures S3 and S4), different Shared Socioeconomic Pathway (SSP) scenarios significantly influence the magnitude and rate of changes in habitat suitability. Across all SSPs, the spatial pattern of suitability from the 2030s to the 2090s exhibits a consistent yet scenario dependent evolutionary trend. Unsuitable areas remain dominant throughout the century, accounting for 82.46–85.70% of the total area, with only minor interdecadal fluctuations. In contrast, low-suitability areas show a continuous expansion under all scenarios and represent one of the most pronounced changes. Their proportion increases from 8.64–10.65% in the 2030s (Figure 10) to 8.48–10.67% in the 2090s (Figure 11), with more evident growth under the SSP370 and SSP585 scenarios. Moderately suitable areas exhibit clear scenario differentiation. Under SSP126 and SSP245, their proportions remain relatively stable across decades, at approximately 3.03–3.17% (Figure 10). Highly suitable areas consistently account for the smallest proportion but display a slight increasing trend across all scenarios. Their share rises from approximately 2.59–2.74% in the 2030s to 2.74–2.91% in the 2090s, with more pronounced increases under the SSP370 and SSP585 scenarios.

In the predicted suitable area of *C. patnalis* (Figure 12 and Figure 13), scenario effects exert a stronger influence on habitat suitability than temporal changes. From the 2030s to the 2090s, the extent and pattern of suitable areas consistently follow scenario-dependent trajectories. The total suitable area is largest in the 2030s (Figure 12), reaches its minimum in the 2050s (Figures S5 and S6), and partially recovers by the 2090s (Figure 13). Unsuitable areas remain dominant throughout the study period, with a variation range within ±2.77%. Low-suitability areas generally decline across all scenarios, with the smallest decrease occurring under SSP126 (−3.09%) and the largest under SSP245 (−13.51%). Moderately suitable areas exhibit pronounced scenario differentiation, showing the greatest increase under SSP585 (+14.99%). Highly suitable areas display the strongest scenario-dependent divergence, with a substantial decrease under SSP245 (−42.18%) and marked increases under SSP370 (+11.15%) and SSP585 (+21.10%).

## 4. Discussion

The occurrence and outbreak intensity of the *C. medinalis* and *C. patnalis* are strongly affected by climatic conditions, source populations, and cropping practices, often resulting in large-scale damage to rice foliage. With the northward shift in rice cultivation areas, the distribution range of these pests had also expanded toward higher latitudes [48]. Understanding future changes in high risk areas is critical for effective pest management. Using MaxEnt modeling combined with field monitoring data and climatic variables, this study characterized the occurrence patterns of *C. medinalis* and *C. patnalis* in Hainan Province and projected their potential suitable habitats globally. These results provide a scientific basis for assessing invasion risk and developing future management strategies under changing environmental conditions.

High-altitude light trap monitoring showed that *C. medinalis* and *C. patnalis* in Hainan had largely synchronized occurrence periods, with peak activity from April to June (Figure 5), coinciding with the booting–heading stages of rice and the most severe damage. Pronounced regional differences in population density were observed, with the highest peaks in Dongcheng Yi and Beishan villages, suggesting that local cropping systems and ecological conditions favor leaf folder development. This pattern is consistent with previous studies showing that changes in rice cultivation practices modify planting patterns and phenology, creating continuous niches that promote pest persistence and complicate monitoring and control [49]. In addition, earlier research has shown that rice leaf folders migrating southward from Guangxi in mid-October along northeasterly airflow may contribute to population fluctuations [50], which corresponds well with the bimodal annual population trends observed in Dongcheng Yi and Beishan villages in this study. Interannual population fluctuations of both species generally followed a unimodal pattern. Genetic analyses revealed distinct differences in the mitochondrial *COI* gene fragment, confirming that *C. medinalis* and *C. patnalis* are closely related yet distinct species. Previous studies suggest that *C. medinalis* and other rice leaf folder species exhibit temporal niche differentiation but substantial spatial overlap when occurring sympatrically [47]. Whether the observed changes in population abundances of *C. medinalis* and the broad-striped leaf folder are driven by spatial interspecific competition following the latter’s immigration remains unclear and warrants further investigation.

The performance of the optimized MaxEnt model was excellent in this research. After the optimization, the model parameters were set as RM=2, FC=L, $AUC_{\mathrm{diff}\left(\mathrm{avg}\right)}=0.066$, and $\Delta AIC_{c}=0$. Notably, the AUC test showed that the accuracy of the model was excellent and could be used for the prediction and analysis of the potentially suitable distribution for *C. medinalis* and *C. patnalis* [51]. This study provides the first prediction of potential suitable habitats for *C. patnalis*, extending previous work on *C. medinalis* and enhancing our understanding of major rice leaf rolling pests. However, as with most species distribution models, limitations remain. The MaxEnt predictions were primarily based on climatic variables, without incorporating biotic factors (e.g., natural enemies) or other abiotic and anthropogenic influences such as soil conditions, topography, and human activities. Consequently, discrepancies between predicted suitable areas and actual occurrence regions may arise due to the combined effects of multiple drivers [45,52,53,54,55].

Clarifying the relationships between insects and environmental variables is essential for understanding the ecological requirements and spatial distribution patterns of insect species [40]. Previous studies have demonstrated that temperature and precipitation are the dominant climatic factors affecting the growth and distribution of the *C. medinalis*, which is consistent with its biological characteristics. In particular, temperature plays a fundamental role in shaping insect survival, development, and reproductive performance. Temperature variability, particularly under warming conditions, may extend the period of pest damage and modify both behavioral traits and outbreak dynamics [33]. Optimal larval performance of *C. medinalis* occurs at 22–28 °C with relative humidity exceeding 80%; however, brief high-temperature exposure may disrupt mating activity [37]. As temperature increases and precipitation patterns shift, the outbreak dynamics of *C. medinalis* may vary among regions, underscoring the importance of developing region specific control strategies [56]. The evaluation results showed that *C. medinalis* was more abundant in areas with high precipitation and low diurnal temperature variation, with seasonal population dynamics. Precipitation in the wettest month was the most influential shared predictor for both species, reflecting a temporal match between larval feeding stages and peak moisture availability. High precipitation supports rice growth, increases tender leaf tissues, and reduces desiccation stress, all of which favor larval survival. Thus, surveillance and integrated control efforts should be prioritized in warm, humid rice-producing regions with high precipitation.

MaxEnt results showed that the current potential suitable areas of *C. medinalis* and *C. patnalis* largely encompass their known occurrence ranges, with Asia and Europe remaining the primary suitable regions under both current and future climates. In China, the northern limit of *C. medinalis* suitability was consistent with previous predictions, whereas *C. patnalis* exhibited a markedly smaller suitable range. Climate change is expected to substantially alter the distributions of both species, as global mean temperature is projected to increase by 1.1–6.4 °C by the end of the 21st century [44]. Our results suggest that suitable habitats for both species will expand markedly, with increases in highly suitable areas in northern and coastal Africa and Australia. The future distribution of *C. patnalis* appears to be driven by warming, changing precipitation patterns, and northward host expansion. Notably, its highly suitable habitat peaked under the SSP245 scenario in the 2030s (2.87%; Figure 12), indicating an elevated risk of cross-regional spread linked to agricultural expansion and international rice seed trade [19]. Overall, these findings suggest that global warming will drive substantial shifts in the extent and distribution of suitable habitats for *C. medinalis* and *C. patnalis*, with continued expansion toward higher latitudes and elevations. Accordingly, enhanced surveillance and management of these major rice pests should be prioritized in key rice-producing regions to safeguard food security and sustainable production.

MaxEnt predictions suggest that suitable areas for *C. medinalis* and *C. patnalis* may deviate from actual distributions due to environmental differences. Annual mean temperature was a key predictor for *C. patnalis*, highlighting its dependence on thermal conditions, particularly in tropical and subtropical regions. In contrast, *C. medinalis* appears more sensitive to seasonal temperature variation and extreme events. Additionally, the model included only 19 bioclimatic variables, excluding biotic factors like human disturbance and natural enemy pressure, which may also affect distribution [45,52,53,54,55]. Accordingly, integrating key biotic and abiotic variables into future models is necessary to obtain more accurate predictions of the potential distributions of *C. medinalis* and *C. patnalis*.

Based on our findings, strengthened control of *C. medinalis* should be coupled with measures to prevent the invasion and spread of *C. patnalis*. Monitoring stations should be established in major rice-producing regions, and a hierarchical early-warning system integrating pest dynamics, biological traits, meteorological factors, and surveillance data should be developed. Optimizing rice cropping systems, enhancing ecological regulation, and promoting green control measures (e.g., pheromone trapping, biological control, and *Bt*-based products) will help reduce reliance on chemical control. In parallel, further research on *C. patnalis* biology and improved identification training are needed to support timely interventions. Overall, pest management should shift from static control to an integrated framework based on dynamic monitoring, ecological regulation, and coordinated regional prevention to ensure sustainable rice production.

## Data Availability

The original contributions presented in this study are included in the article/Supplementary Materials. Further inquiries can be directed to the corresponding authors.

## Supplementary Materials

The following supporting information can be downloaded at: https://www.mdpi.com/article/10.3390/insects17010126/s1, Table S1. Climate variables and their contributions; Table S2. Evaluation metrics during the tuning process; Figure S1. Pearson correlation analysis and correlation coefficients of 19 bioclimatic variables; Figure S2. The AUC values of the *C. medinalis* and *C. patnalis*; Figure S3. Distribution prediction of *C. medinalis* under different climate scenarios for the 2050s; Figure S4. Distribution prediction of *C. medinalis* under different climate scenarios for the 2070s; Figure S5. Distribution prediction of *C. patnalis* under different climate scenarios for the 2050s; Figure S6. Distribution prediction of *C. patnalis* under different climate scenarios for the 2070s; Figure S7. Jackknife test gain for *C. medinalis* and *C. patnalis*.
