# Supplementary material for: Integrating Morphological, Molecular, and Climatic Evidence to Distinguish Two Cryptic Rice Leaf Folder Species and Assess Their Potential Distributions
Source: Insects. 2026 Jan 22;17(1):126. doi: 10.3390/insects17010126 (PMC12842515; doi:10.3390/insects17010126)
Supplement: Supplementary file 1 [file insects-17-00126-s001.zip › insects-4074259-supplementary.pdf]

Table S1. Climate variables and their contributions.

Code	Environmentsl varisbles	Units	*C. medinalis*		*C. patnalis*	
			Percent contribution	Permutation importance	Percent contribution	Permutation importance
BIO 01	Annual Mean Temperature	℃	0	0	4	26
BIO 02	Mean Diurnal Range (Mean of monthly (max temp - min temp))	℃	0.5	3.8	6.7	6.6
BIO 03	Isothermality (BIO 02/BIO 07) (×100)	%	11.1	12.7	5.6	1.6
BIO 04	Temperature Seasonality (standard deviation*100)	%	3.2	2.7	1.7	6.9
BIO 05	Max Temperature of Warmest Month	℃	0.4	1.3	0	1
BIO 06	Min Temperature of Coldest Month	℃	1.8	1	0.8	10.1
BIO 07	Temperature Annual Range (BIO 05-BIO 06)	℃	0.8	1.1	0.8	0.1
BIO 08	Mean Temperature of Wettest Quarter	℃	3.9	15.1	0	3
BIO 09	Mean Temperature of Driest Quarter	℃	0.1	0	1	5.7
BIO 10	Mean Temperature of Warmest Quarter	℃	1.9	15.3	3.8	2.1
BIO 11	Mean Temperature of Coldest Quarter	℃	1.8	0	22.9	17.2
BIO 12	Annual Precipitation	mm	0.7	3.6	1.6	2.1
BIO 13	Precipitation of Wettest Month	mm	21.5	23.1	29.8	5.2
BIO 14	Precipitation of Driest Month	mm	0	0	5.4	5.1
BIO 15	Precipitation Seasonality (coefficient of variation)	%	0.6	0.8	2.6	2
BIO 16	Precipitation of Wettest Quarter	mm	42.2	0.1	7.4	1.5
BIO 17	Precipitation of Driest Quarter	mm	0.3	0.4	0.6	1
BIO 18	Precipitation of Warmest Quarter	mm	7.2	14.6	1.3	1.7
BIO 19	Precipitation of Coldest Quarter	mm	2	4.3	3.7	2

Note: Environmental factors of the underlined category indicate those selected as the main variables applied in the model prediction.

Table S2. Evaluation metrics during the tuning process.

FC	*RM*	AUC_{train}	CBI_{train}	$AUC_{diff\ (avg)}$	$AUC_{val\ (avg)}$	$CBI_{val\ avg}$	$OR_{10\%\ (avg)}$	$OR_{MTP\ avg}$	*AICc*	*Delta.AICc*	*wAICc*	*nCoef*
L	1	0.888684	0.954	0.06721245	0.8578939	0.7135	0.1938406	0.1195652	1534.792	5.0687315	4.13E-02	15
LQ	1	0.8927849	0.954	0.07837114	0.8633227	0.66075	0.1843297	0.1304348	1560.409	30.6860554	1.13E-07	19
H	1	0.9048611	0.941	0.05640139	0.8513098	0.72025	0.2160326	0.151721	1606.456	76.7330187	1.13E-17	34
LQH	1	0.9039957	0.96	0.06469041	0.843393	0.55575	0.205163	0.151721	1590.043	60.3198068	4.15E-14	32
**L**	**2**	**0.8866469**	**0.961**	**0.0663711**	**0.862341**	**0.75275**	**0.1621377**	**0.1195652**	**1529.723**	**0**	**5.21E-01**	**12**
LQ	2	0.8908809	0.945	0.06995493	0.86487	0.68925	0.1838768	0.1304348	1561.654	31.9310518	6.06E-08	16
H	2	0.89389	0.944	0.06035962	0.8628285	0.76875	0.1942935	0.1408514	1576.571	46.8474005	3.50E-11	20
LQH	2	0.8966594	0.949	0.0617777	0.858623	0.70975	0.205163	0.1408514	1578.77	49.0466826	1.16E-11	24
L	3	0.884197	0.972	0.06504222	0.8645565	0.74175	0.1621377	0.1195652	1530.091	0.3677204	4.33E-01	9
LQ	3	0.88678	0.97	0.06625951	0.8648424	0.757	0.1730072	0.1304348	1569.648	39.924499	1.11E-09	16
H	3	0.8865004	0.872	0.06318589	0.8658568	0.733	0.1942935	0.1195652	1564.282	34.5583219	1.63E-08	12
LQH	3	0.8876455	0.929	0.06317088	0.8654825	0.6975	0.1942935	0.1304348	1579.08	49.3564757	9.97E-12	20
L	4	0.883265	0.958	0.06504474	0.8653745	0.71175	0.1621377	0.1195652	1539.195	9.4713658	4.57E-03	10
LQ	4	0.8850358	0.969	0.06549473	0.8657523	0.72325	0.1621377	0.1195652	1559.066	29.3422262	2.21E-07	13
H	4	0.8849959	0.909	0.06621481	0.8646166	0.71425	0.1725543	0.1195652	1566.284	36.5606427	5.99E-09	13
LQH	4	0.8854486	0.962	0.06396389	0.8653644	0.69125	0.1621377	0.1195652	1561.696	31.972981	5.94E-08	13
L	5	0.8823729	0.965	0.06483432	0.8653264	0.687	0.1621377	0.1304348	1544.01	14.2867398	4.11E-04	9
LQ	5	0.8837177	0.975	0.06486268	0.8657945	0.71075	0.1621377	0.1304348	1548.349	18.6255332	4.70E-05	9
H	5	0.8823929	0.942	0.06619764	0.8628681	0.695	0.1725543	0.1195652	1553.248	23.5242185	4.06E-06	11
LQH	5	0.8838242	0.971	0.06394909	0.865351	0.68325	0.1621377	0.1304348	1552.509	22.7855778	5.87E-06	10

Note: The parameter combination highlighted in bold was selected for use in the manuscript.

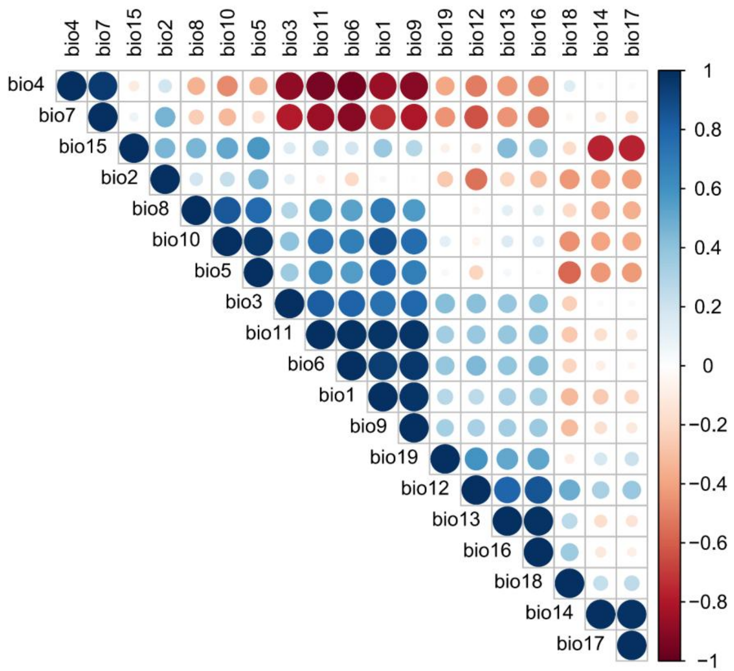

Figure S1. Pearson correlation analysis and correlation coefficients of 19 bioclimatic variables.

Note: The color of red means positive correlation, blue means negative correlation. The size of the circle means the strength of the correlation. Bio1 represents the Annual mean temperature, Bio2 represents the Monthly mean temperature difference, Bio3 represents the Isothermality, Bio4 represents the Temperature seasonality, Bio5 represents the Max temperature of warmest month, Bio6 represents the Min. temperature of the coldest month, Bio7 represents the Temperature annual range, Bio8 represents the Mean temperature of the wettest quarter, Bio9 represents the Mean temperature of driest quarter, Bio10 represents the Mean temperature of warmest quarter, Bio11 represents the Mean temperature of coldest quarter, Bio12 represents the Annual precipitation, Bio13 represents the Precipitation of wettest month, Bio14 represents the Precipitation of driest month, Bio15 represents the Precipitation seasonality, Bio16 represents the Precipitation of wettest quarter, Bio17 represents the Precipitation of driest quarter, Bio18 represents the Precipitation of warmest quarter , Bio19 represents the Precipitation of coldest quarter.

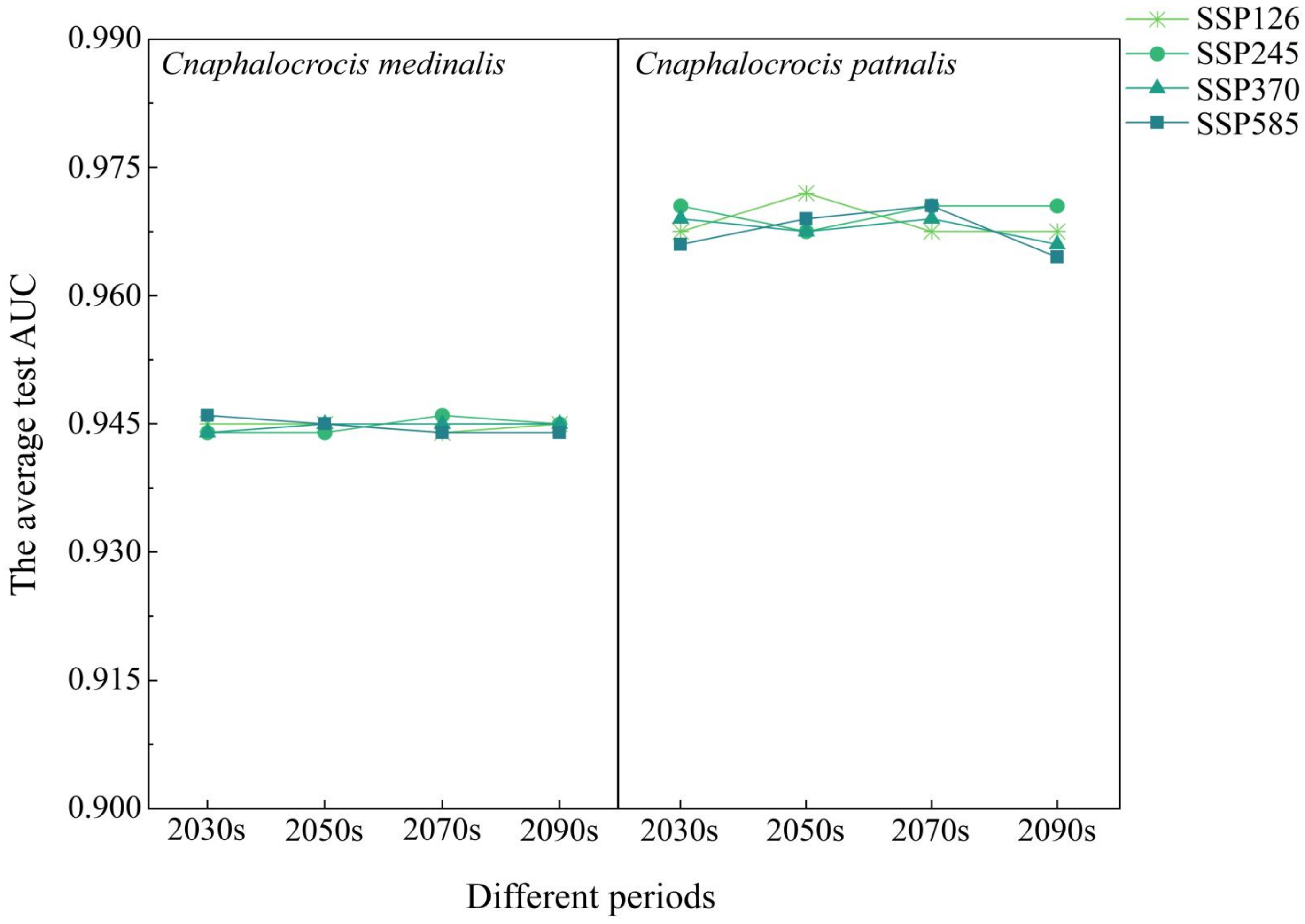

Figure S2. The AUC values of the *C. medinalis* and *C. patnalis*

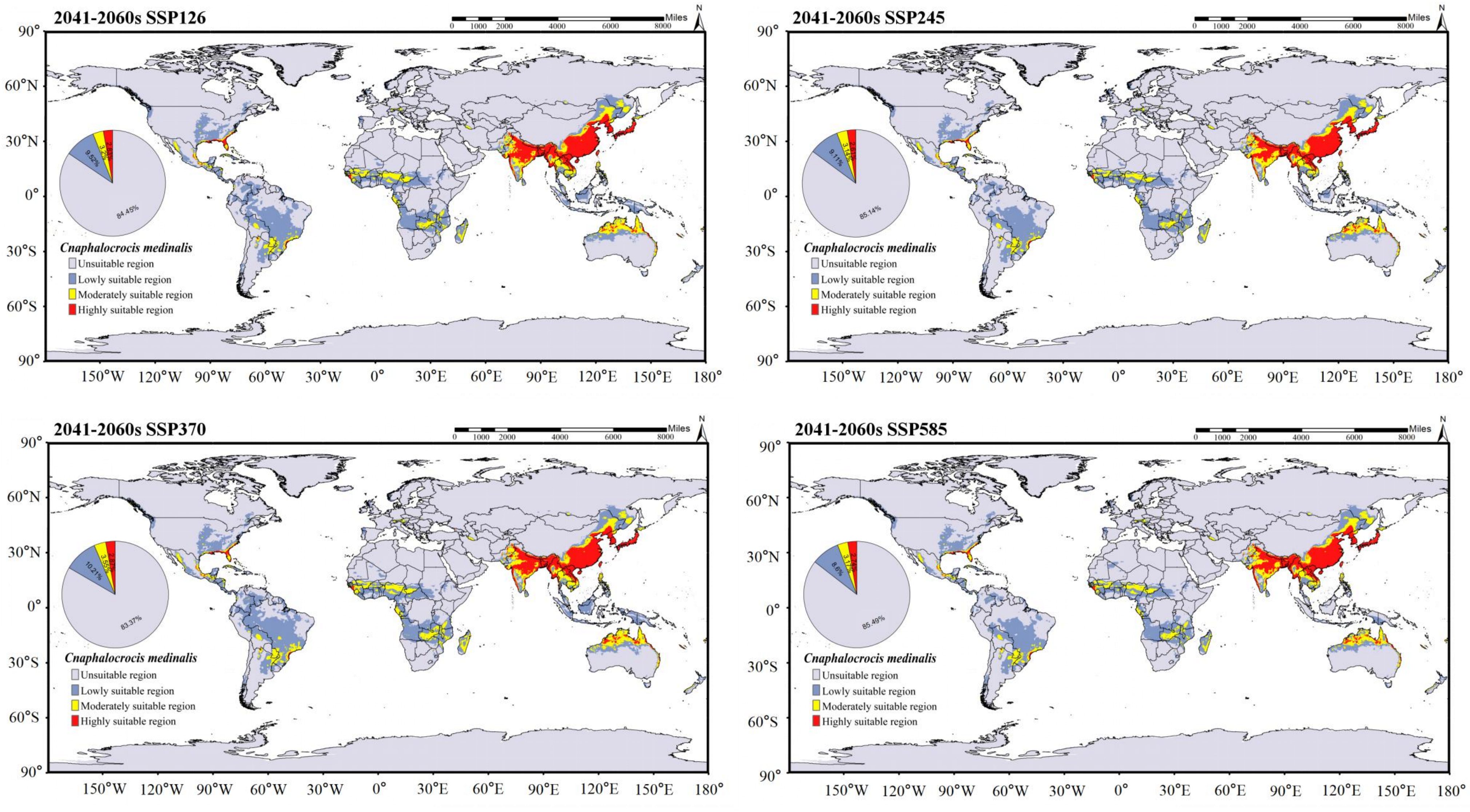

Figure S3. Distribution prediction of *C. medinalis* under different climate scenarios for the 2050s.

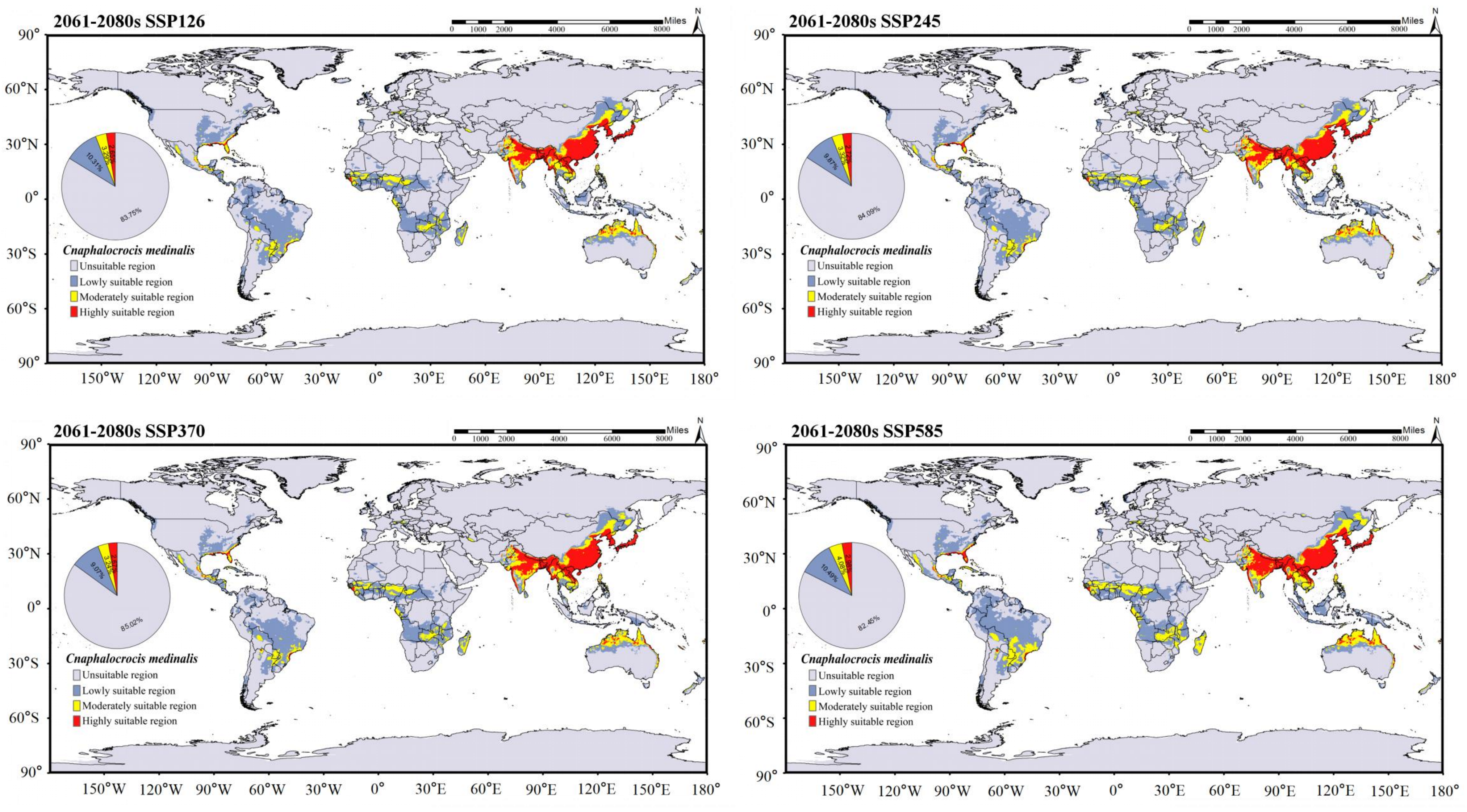

Figure S4. Distribution prediction of *C. medinalis* under different climate scenarios for the 2070s.

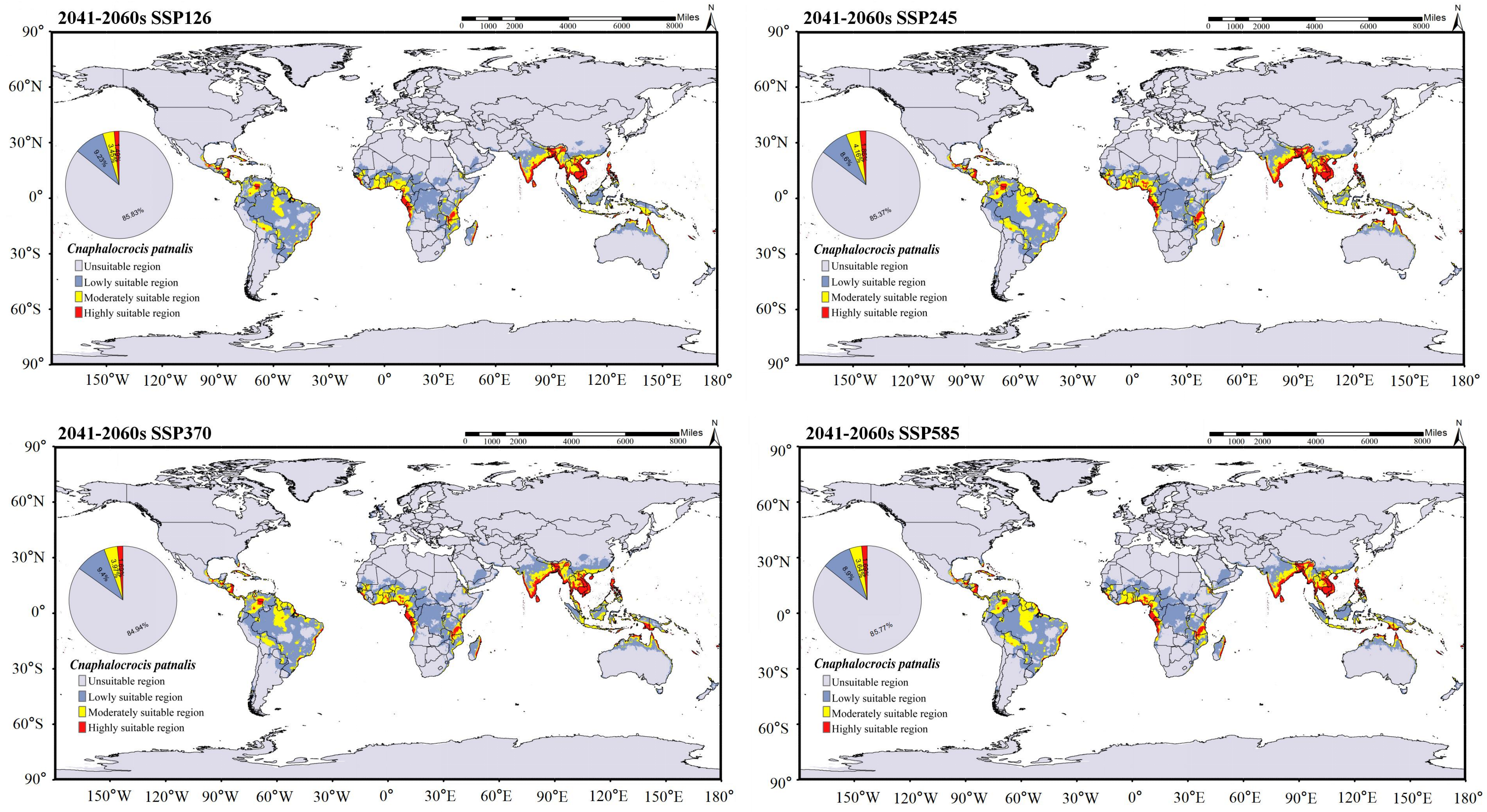

Figure S5. Distribution prediction of *C. patnalis* under different climate scenarios for the 2050s.

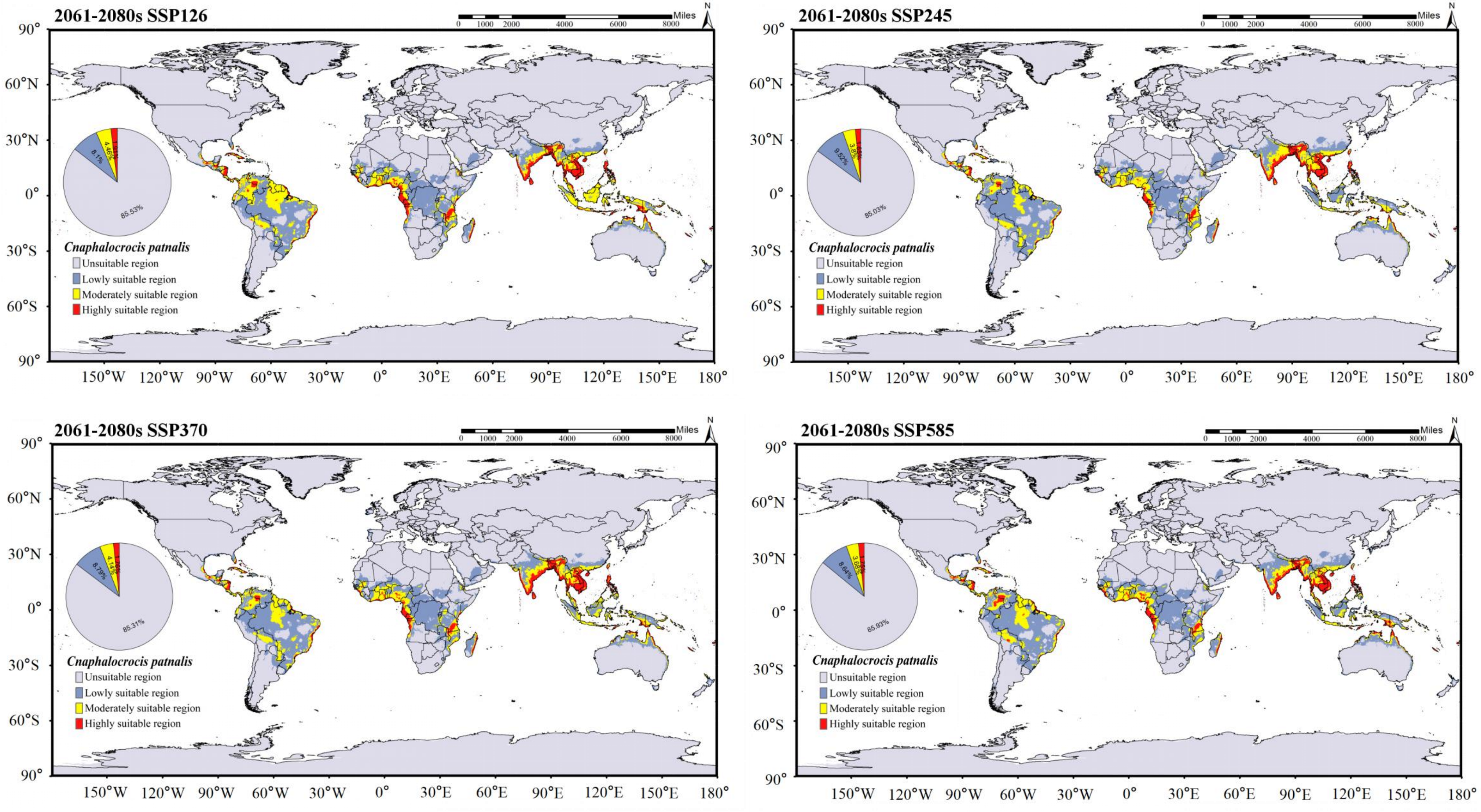

Figure S6. Distribution prediction of *C. patnalis* under different climate scenarios for the 2070s.

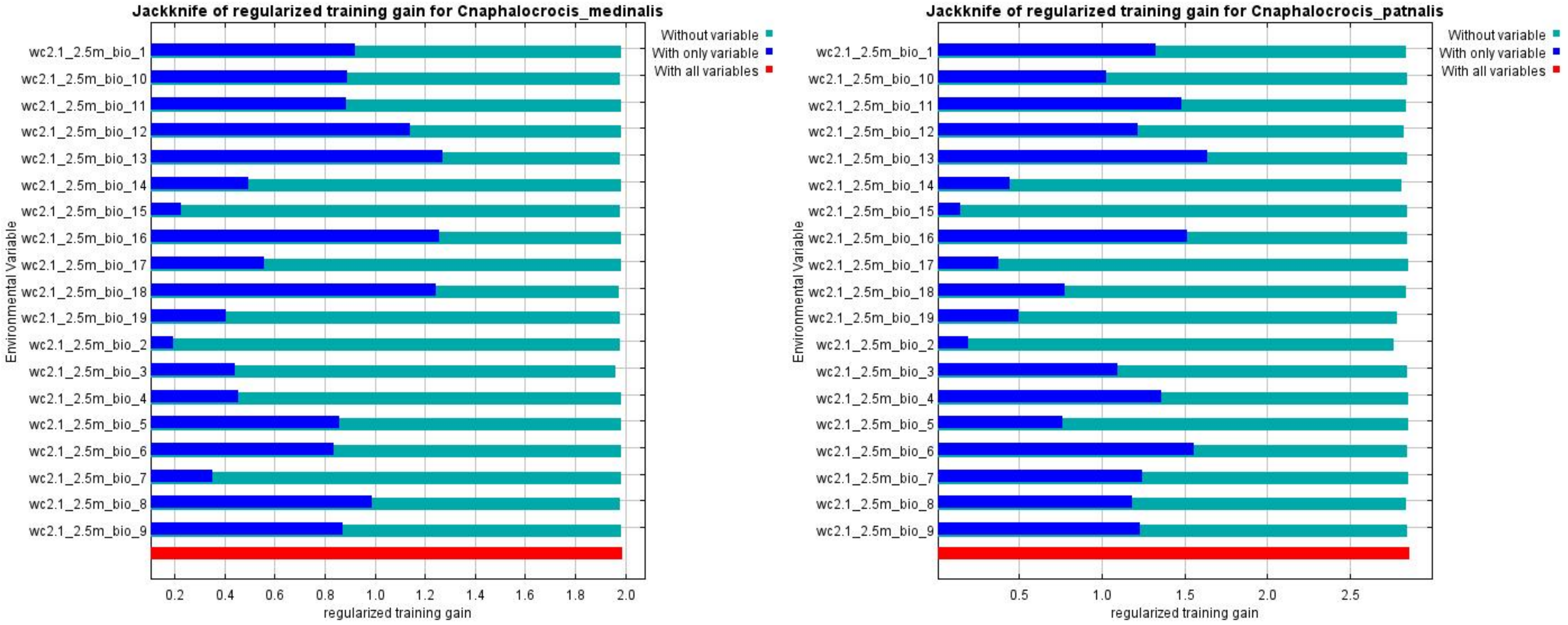

Figure S7. Jackknife test gain for *C.medinalis* and *C.patnalis*. Blue, green, and red bars represent running the MaxEnt model with the variable alone, without the variable, and with all variables, respectively.
